# Cognitive Bias in Action: Delayed Diagnosis of Thoracic Meningioma Mimicking Lumbosacral Radiculopathy—A Case Report

**DOI:** 10.1002/ccr3.70867

**Published:** 2025-09-07

**Authors:** Ji‐Hoon Lee, Bi Mo, Jerry Markar

**Affiliations:** ^1^ David Geffen School of Medicine at UCLA Los Angeles California USA; ^2^ Department of Anesthesiology and Perioperative Medicine, Division of Pain Medicine David Geffen School of Medicine at UCLA Los Angeles California USA

**Keywords:** cognitive bias, epidural, radiculitis, sciatica, thoracic meningioma

## Abstract

The highly variable clinical presentations of sciatica, combined with cognitive biases, often lead to misattribution of the underlying pathology responsible for patient symptoms. Such limitations can contribute to significant diagnostic delays. Prioritizing systematic evaluation and maintaining vigilance against biases are critical for ensuring timely diagnosis and improving patient outcomes.

## 1 Introduction

Pain radiating from the spine to the leg is commonly referred to as “sciatica.” Patients with sciatica typically present with radicular pain, with or without accompanying radiculopathy. The pain is often described as sharp, burning, and radiating along the distribution of the sciatic nerve. It may also be associated with other neurological symptoms, including numbness, tingling, or weakness [1, 2, 3].

Sciatica is most commonly caused by mechanical irritation or compression of one or more lumbar nerve roots. Lumbar disc herniations secondary to degenerative disc disease (DDD), particularly at the L4–L5 and L5–S1 levels, account for over 90% of cases, representing the most common etiology of sciatica [1]. The majority of acute sciatica cases due to lumbar disc herniation are self‐limiting, with symptoms resolving within weeks to a few months in 70% to 90% of patients [1, 2]. First‐line treatment is conservative management and includes nonsteroidal anti‐inflammatory drugs (NSAIDs), physical therapy (PT), and, when indicated, prescription analgesics [3]. Adjunctive therapies such as chiropractor care, massage therapy, and acupuncture may offer symptomatic relief for some patients [4, 5, 6].

When pain does not improve or symptoms include neurological deficits, patients should undergo advanced imaging (e.g., computed tomography [CT] or magnetic resonance imaging [MRI]) to assess for underlying structural abnormalities. In the absence of severe spinal cord or cauda equina compression, epidural steroid injections (ESIs) may be considered to alleviate inflammation and radicular pain [7]. However, patients with progressive neurological symptoms or imaging evidence of significant neural element compression should be referred for neurosurgical evaluation [8].

When conventional treatments fail to relieve symptoms or the clinical picture evolves, clinicians should expand the differential diagnosis to include rarer causes of radicular pain. These include herpes zoster, sacroiliitis, piriformis syndrome, intrapelvic masses, and spinal neoplasms [9, 10]. Rare extraspinal causes, such as infection, piriformis syndrome, and gynecologic conditions, may be considered [9, 10]. Diagnosing these atypical etiologies requires a high index of suspicion and a tailored diagnostic approach based on history, examination, and imaging findings. Definitive treatment hinges on accurately identifying the underlying cause and initiating targeted management.

In this case report, we describe a patient who was ultimately diagnosed with a thoracic meningioma presenting with sciatica‐like symptoms. The initial symptoms were attributed to a small foraminal disc protrusion at L4–L5, identified on early imaging. However, after months of unsuccessful conservative therapy, multiple rounds of ESIs, and acute onset neurological deficits, further imaging revealed a thoracic spinal tumor as the true etiology. This case highlights the importance of maintaining a broad differential diagnosis and exercising caution against cognitive biases such as premature diagnostic closure—particularly when a patient's clinical trajectory diverges from the expected course.

## 2 Case Presentation

### 2.1 Clinical Course

A 50‐year‐old female with no significant past medical history presented with low back pain radiating into the left lower extremity (LLE), which began 8 months prior to initial presentation. The pain was partially managed with home exercises, yoga, acupuncture, and stretching. However, the pain acutely worsened a couple of weeks before presentation, rated at 7/10, particularly intense at night and when lying down. The patient reported no significant relief with ibuprofen. At this stage, her daily activities were limited due to pain, though she denied any neurological symptoms such as tingling, numbness, or weakness. She was started on gabapentin 300 mg daily, which provided effective relief for approximately two months. Concurrently, she also initiated PT. The patient, on consultation with Physical Medicine and Rehabilitation, received lumbar MRI, which showed left foraminal disc protrusion at L4‐5.

Despite these interventions, she began experiencing intermittent tingling in her left foot. By the third month of initial presentation, the patient's pain worsened to 10/10, severely impairing sleep. A repeat lumbar MRI showed no progression from the prior scan. The patient subsequently underwent a left L4–L5 and L5–S1 transforaminal ESI (TFESI), which provided transient relief. Over the following months, pain persisted and escalated, leading to two additional L4–L5 interlaminar ESIs (ILESI), spaced two months apart. The first injection offered temporary relief for 36 h, while the second resulted in > 50% improvement lasting over three months before recurrence. A third L4–L5 ILESI, six months after the last, was performed, which provided only 48 h of benefit. Given the lack of significant improvement with non‐operative management, she was referred for surgical consultation but was deemed not a surgical candidate due to the absence of high‐grade central or neuroforaminal stenosis on MRI. Electromyography (EMG) and nerve conduction studies (NCS) were recommended for further evaluation but demonstrated unremarkable results.

Fifteen months after the initial presentation, the patient was referred to our pain clinic for further evaluation. At that time, she remained neurologically intact, with normal strength, sensation, and deep tendon reflexes. Given the lack of improvement following multiple ESIs, a piriformis muscle steroid injection was administered, but it failed to provide meaningful relief. The patient also received one round of caudal ESI (CESI). She was subsequently referred to neurology for further evaluation to rule out other causes of her persistent LLE pain. However, she delayed the neurology consultation due to transient symptom improvement.

By the time she was evaluated by neurology, her symptoms had acutely worsened. In addition to increased LLE pain, she had developed new right lower extremity (RLE) weakness. She reported that her right leg frequently “gave out” and noted numbness in the same limb during this period. She had experienced 4–5 falls attributed to right leg weakness, including three within the prior three weeks. Notably, she denied pain in the right leg but described increasing difficulty with balance.

Neurological examination revealed upper motor neuron signs, including 3+ hyperreflexia at the knees and ankles bilaterally. She also exhibited a 50% decrease in pinprick sensation on the right side below the T4 dermatome, while vibration sensation remained intact. A positive Romberg test indicated impaired proprioception, and her gait was wide‐based with evident dragging of the right leg. Additionally, she was unable to rise from a chair without using her arms for assistance. These concerning findings prompted an urgent MRI of the thoracic spine, which ultimately revealed the underlying diagnosis.

### 2.2 Investigation and Treatment

**FIGURE 1:**
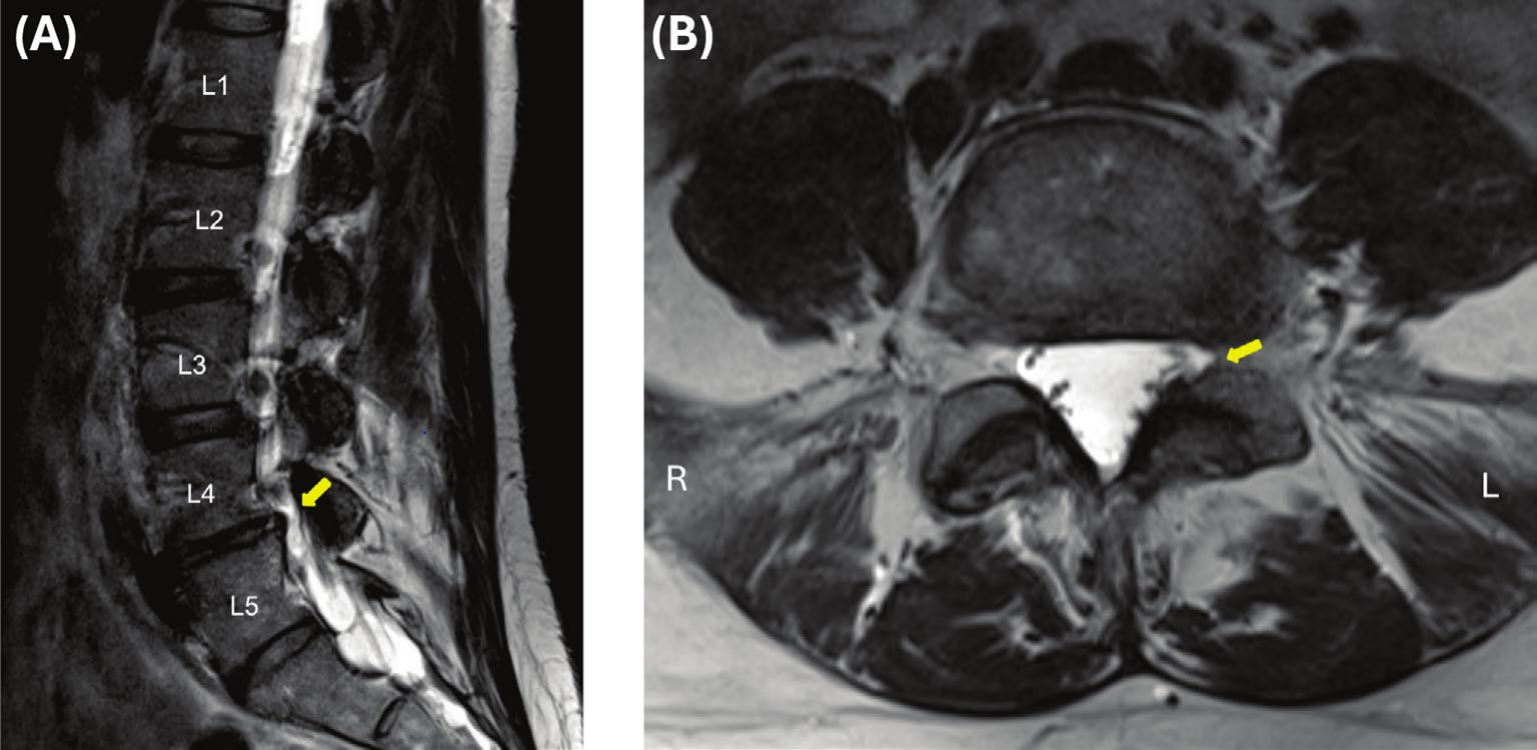
MRI of the lumbar spine without contrast, highlighting disc degeneration and disc height loss at L4–L5, accompanied by a small left foraminal disc protrusion that resulted in mild narrowing of the left neural foramen (Yellow Arrows) in (A) sagittal and (B) axial planes.

**FIGURE 2:**
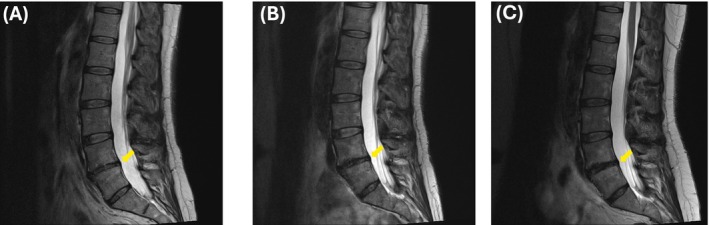
MRI of the lumbar spine without contrast, highlighting disc degeneration (Yellow Arrows) at the L4–L5 level with mild left facet arthropathy and mild ligamentum flavum thickening. Images acquired in (A) June 2024, (B) November 2022, and (C) March 2024 demonstrate no significant temporal progression.

On lumbar MRI, there was disc degeneration and disc height loss at L4–L5, accompanied by a small left foraminal disc protrusion that resulted in mild narrowing of the left neural foramen (Figure 1). Repeat lumbar MRI studies revealed no gross temporal discogenic degenerative changes at the L4–L5 level (Figure 2). These findings led to the attribution of the degenerative discogenic changes at L4–L5 as the potential cause of the patient's symptoms, and she was treated accordingly as described in her clinical course.

EMG and NCS were performed and revealed normal results. There was no evidence of lumbar radiculopathy in the left lower extremity, as the tested muscles did not show signs of denervation or reinnervation. Moreover, there was no indication of nerve entrapment (e.g., tarsal tunnel syndrome) or generalized peripheral neuropathy in the bilateral lower extremities.

Over the course of treatment, the patient received numerous epidurals (1 TFESI, 3 ILESI, 1 CESI) and a piriformis injection. Unfortunately, these interventions provided only minimal and transient relief, with improvements lasting no more than 48 h before her pain returned.

**FIGURE 3:**
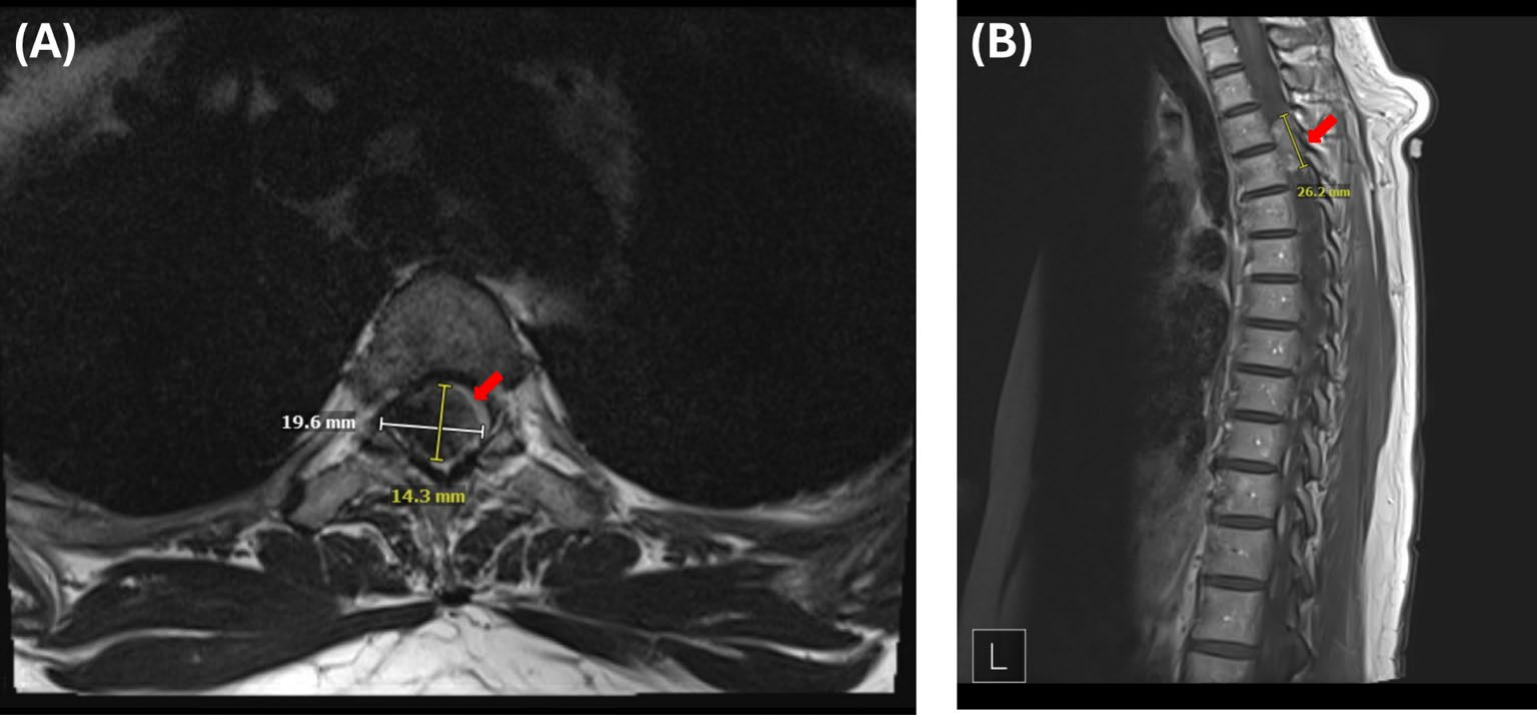
Pre‐operative MRI of the thoracic spine with and without contrast, showing an approximately 2.6 × 2.0 × 1.4 cm calcified mass at the T3–T4 level, consistent with a benign meningioma (Red Arrows). Images are presented in (A) axial and (B) sagittal planes.

The urgent thoracic spine MRI revealed a 1.4 × 2.0 × 2.6 cm calcified mass at the T3–T4 level (Figure 3). The mass was compressing the thecal sac contents, including the spinal cord, and was associated with compressive myelopathy. The patient was subsequently referred to neurosurgery and underwent a T2–T5 laminectomy and fusion with extramedullary intradural tumor resection. Pathology confirmed the diagnosis of benign meningioma of the psammomatous subtype.

### 2.3 Outcome and Follow‐Up

The patient tolerated the operation well, with no perioperative complications, and was admitted to the intensive care unit (ICU) in stable condition for standard postoperative management and monitoring. By day 1 of surgery, the patient reported resolution of preoperative weakness and numbness, with expected postoperative back pain. By day 2, the patient was ambulating independently and maintaining a stable neurologic exam. Pain was adequately controlled with oral medications, and the patient was subsequently discharged with recommendations for home PT and oral pain medications.

**FIGURE 4:**
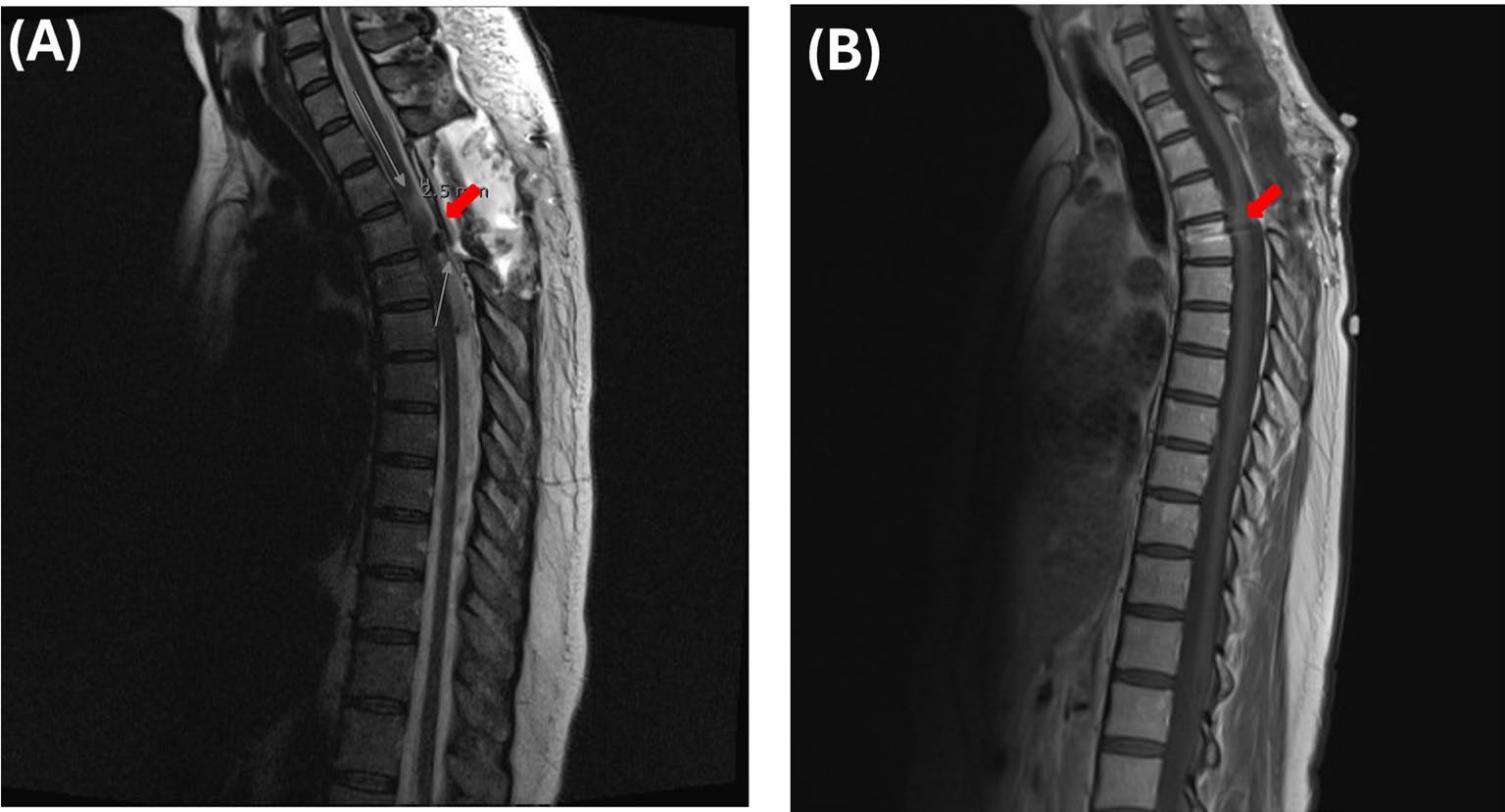
(A) Post‐operative, and (B) most recent follow‐up MRI of the thoracic spine with and without contrast, demonstrating no residual lesion at the T3–T4 level (Red Arrows).

Post‐operative MRI revealed no evidence of residual mass, with appropriate T2–T5 posterior spinal instrumentation and fusion (Figure 4A). New postsurgical changes included findings consistent with T3–T4 laminectomies and tumor resection, with near‐complete resolution of cord compression. Residual T2 hyperintensity was observed at the T2–T3 spinal cord level, corresponding to the previous site of compression.

The patient has since been recovering well through home rehabilitation and reported complete resolution of her myelopathy symptoms, with stable MRI findings (Figure 4B). At the most recent follow‐up, she noted a successful return to work.

## 3 Discussion

Despite extensive clinical trials aimed at establishing a standardized treatment course for sciatica, no universally accepted approach has been identified as superior. Nevertheless, the guidelines developed by the American College of Physicians (ACP) and the American Pain Society (APS) provide a comprehensive framework by synthesizing a broad spectrum of evidence into actionable recommendations [11]. These guidelines evaluate various treatment modalities for low back pain, ranking them based on their expected net clinical benefits.

The diagnostic complexity of sciatica increases significantly in cases where atypical etiologies mimic sciatica through false‐localizing mechanisms. For example, neoplastic lesions in the thoracic or cervical spine may present with symptoms resembling lumbar sciatica [12, 13, 14, 15, 16, 17, 18], often leading to prolonged diagnostic delays. These delays can have profound implications for patient outcomes, particularly in cases of malignancy, where early intervention may significantly alter prognosis. Kato et al. highlighted the variability in diagnostic delays for spinal tumors, reporting averages of 16.9, 16.8, and 8.1 months for cervical, thoracic, and lumbar tumors, respectively [19]. Such delays frequently arise from overlapping clinical features of sciatica and other intra‐ or extra‐spinal pathologies, compounded by incidental imaging findings. We conducted an updated literature review, incorporating case reports of spinal neoplasms not included in the summaries provided in [14, 20] (Table 1). Non‐neoplastic etiologies, such as sacroiliitis, piriformis syndrome, and discogenic conditions [10], were excluded from the review.

**TABLE 1:** Rare etiology of cervical and thoracic pathology mimicking sciatica.

Case#	Diagnosis	Spinal level	TTD	Indication	Ref
This case	Meningioma	T3–T4	30 months	Acute onset neurological deficits	—
2	Meningioma	T10	4 months	Incidental	[15]
3	Meningioma	T11	Acute	Acute onset neurological deficits after trauma	[16]
4	Schwannoma	T5	6 months	No improvement post laminectomy and discectomy	[14]
5	Schwannoma	T9–T10	10 years	6 months of worsening neurological symptoms	[17]
6	Schwannoma	C1–C2	3 years	Progressively worsening neurological symptoms	[18]

Abbreviation: TTD, time‐to‐diagnosis from onset of symptoms.

An additional layer of diagnostic complexity arises from misattribution of imaging findings, particularly in patients whose lumbar abnormalities are incorrectly assumed to be the root cause of their symptoms. In this case, the patient exhibited radiologic findings suggestive of left foraminal stenosis at L4–L5. However, the true etiology of her symptoms was ultimately identified as a thoracic meningioma, diagnosed 22 months after the initial presentation and following multiple unsuccessful steroid injection interventions. This underscores the importance of maintaining a broad differential diagnosis and considering less common etiologies when symptoms are refractory to conventional treatments.

Cognitive biases, particularly anchoring bias, may have contributed to the diagnostic delay observed in this case. Anchoring bias occurs when clinicians fixate on initial findings, such as lumbar imaging abnormalities, and prematurely conclude that these findings explain the patient's symptoms. This premature diagnostic closure can preclude consideration of alternative diagnoses, including rarer pathologies such as neoplasms in the thoracic or cervical spine. Similarly, confirmation bias—interpreting subsequent evidence to fit the initial diagnosis—may have further perpetuated the delay. For instance, multiple rounds of ESI were pursued despite a lack of sustained improvement, influenced, at least in part, by these biases in clinical decision‐making.

To address these challenges, updated guidelines should emphasize the need for a systematic approach to diagnostic workup, particularly in cases where symptoms are refractory to treatment or imaging findings do not correlate well with clinical presentation. Moreover, guidelines should incorporate protocols to minimize cognitive biases in clinical practice.

## Author Contributions

**Ji‐Hoon Lee:** conceptualization, data curation, formal analysis, investigation, methodology, project administration, writing – original draft, writing – review and editing. **Bi Mo:** supervision, validation, writing – original draft, writing – review and editing. **Jerry Markar:** conceptualization, investigation, methodology, resources, supervision, validation, writing – original draft, writing – review and editing.

## Ethics Statement

Per UCLA policy, a case report that complies with HIPAA does not require IRB approval.

## Consent

Written informed consent was obtained from the patient for the publication of this case report and accompanying images. A copy of the signed consent is available for review upon request.

## Conflicts of Interest

The authors declare no conflicts of interest.

## Data Availability

Data and materials utilized to prepare for this case report were drawn from the patient's electronic medical records. Patient's protected health information is not available for disclosure per HIPAA.
